# Downregulated ESRP1/2 promotes lung metastasis of bladder carcinoma through altering FGFR2 splicing and macrophage polarization

**DOI:** 10.3389/fimmu.2023.1161273

**Published:** 2023-04-05

**Authors:** Yuyang Zhao, Mingyang Li, Wenbo Wu, Wenhao Miao, Haitao Liu

**Affiliations:** Department of Urology, Shanghai General Hospital, Shanghai Jiao Tong University School of Medicine, Shanghai, China

**Keywords:** Bladder carcinoma, ESRP1, ESRP2, FGFR2, splicing, tumor-associated macrophages, macrophage polarization

## Abstract

**Introduction:**

Lung metastasis occurs in parts of the bladder carcinoma (BC) patients but represents the highest severity and a poor outcome of the disease. The molecular mechanism underlying lung metastasis of BC is not fully understood. Fibroblast growth factor receptor 2 (FGFR2) signaling plays a substantial role in the BC cell growth and invasion. In this study, we assessed the regulation of the alternative splicing of FGFR2 by epithelial splicing regulatory proteins (ESRPs) in lung metastasis of BC.

**Methods:**

Gene profile of BC in comparison with adjacent non-tumor bladder tissue was obtained from GEO public database to analyze the levels of differentiated genes and pathways. Moreover, the association of ESRP1 or ESRP2 with lung metastasis of BC was analyzed on our own clinic samples. The effects of altered expression of ESRP1 or ESRP2 on alternative splicing of FGFR2 IIIb and IIIc, which represents epithelial and mesenchymal-like splicing, were analyzed on BC cell lines T24 and RT4. The *in vivo* effects of ESRP1 or ESRP2 on lung metastasis of BC were assessed in mice subcutaneously grafted with ESRP1/2-modified BC labeled with fluorescent and luciferase reporters.

**Results:**

We detected significant reduction of ESRP1 and ESRP2 in BC in public database of BC specimens. Moreover, analysis on our own specimens also showed strong downregulation of ESRP1 or ESRP2 in BC, and the latter was more pronounced in cases with lung metastasis. *In vitro*, altered levels of ESRP1 or ESRP2 caused a switch of FGFR2 splicing between FGFR2-IIIb and FGFR2-IIIc, resulting in changes in tumor cell growth and metastatic potential. *In vivo*, re-expression of ESRP1 or ESRP2 in BC cells not only inhibited the growth of the xenografted tumor formation in nude mice, but also reduced the occurrence of lung metastasis, partially through altering polarization of tumor-associated macrophages.

**Conclusion:**

Our data thus suggest that reduction in ESRP1 or ESRP2 promotes lung metastasis of BC through altering FGFR2 splicing and macrophage polarization.

## Introduction

Among all common noncutaneous malignancies, bladder carcinoma (BC) is the fourth prevalent one in all patients from the United States, and a subset of BC can progress to a severe muscle invasive form, leading to distal metastasis to other organs, including liver, lung, bone and mediastinum (1). BC with lung metastasis typically have poor outcome and 5-year survival of the patients. Specifically, lung metastasis of BC occurs in parts of the patients but represents the highest severity of the disease (2). Epithelial-to-mesenchymal transition (EMT) allows the epithelial cells to increase their migratory and invasive potential to become mesenchymal cells through losing cellular polarity and cell-cell adhesion (3). EMT is the key step in the initiation of cancer metastasis, and it allows the tumor cells to detach from the mass, invade peripheral tissue and even move to distal organs, like lung (4). However, the molecular mechanism underlying lung metastasis of BC is not fully understood (5–7).

Fibroblast growth factor receptor 2 (FGFR2) signaling plays a substantial role in the BC cell growth and invasion (8). Alternative splicing is a process that mediates differential insert of exons into a mature mRNA transcript to allow generation of different mRNAs from a single gene and to result in producing distinct protein isoforms (9). There are two major splicing isoforms of FGFR2, known as FGFR2IIIb and FGFR2IIIc, that differ in their extracellular domains (9). The FGFR2IIIb isoform is primarily expressed in epithelial cells, while FGFR2IIIc is more common in mesenchymal cells (9). Alternative splicing of the FGFR2 gene produces various isoforms that exhibit distinct properties in terms of ligand binding and downstream signaling (9). Recently, epithelial splicing regulatory proteins (ESRPs) have been shown to regulate the alternative splicing of FGFR2 to affect EMT-associated cancer cell metastasis in pancreatic cancer (10), non-small cell carcinoma (11, 12) and colorectal carcinoma (13). However, a role of ESRPs in BC-associated lung metastasis has not been studied. Moreover, the mechanism was unknown.

The alternative splicing of FGFR2 has been implicated in affecting macrophage polarization, which refers to the process by which macrophages can switch between different functional states in response to signals in their microenvironment (14). Macrophages are a type of immune cell that can adopt either a pro-inflammatory or anti-inflammatory phenotype, depending on the signals they receive (15). Pro-inflammatory macrophages (also known as M1 macrophages) are activated in response to infectious agents or tissue damage and play a role in the clearance of pathogens and promotion of tissue repair (16). Anti-inflammatory macrophages (also known as M2 macrophages) are activated in response to signals such as cytokines, growth factors, and metabolic changes, and are thought to play a role in tissue remodeling, angiogenesis, and suppression of immune responses (17). TAMs are more M2-like macrophages (17). Studies have shown that alternative splicing of FGFR2 can impact the polarization of macrophages, by altering the expression and activity of the receptor (14).

Tumor-associated macrophages (TAMs) are a type of immune cell that are commonly found in the microenvironment of many types of solid tumors (18). TAMs are thought to play a complex role in the progression of cancer, with some studies suggesting that they can both promote and suppress tumor growth (19). TAMs can be activated by cytokines and growth factors produced in the tumor microenvironment (18). Once activated, TAMs can secrete various cytokines and growth factors that can promote angiogenesis, immune suppression, and other processes that contribute to tumor growth and progression (18). TAMs have been implicated in promoting tumor metastasis through several mechanisms. These include promoting angiogenesis, creating an immune-suppressive microenvironment, breaking down the extracellular matrix, and providing direct support to cancer cells by promoting their migration and invasion into surrounding tissues. TAMs secrete cytokines and growth factors that can promote the growth of new blood vessels, which helps create a supportive microenvironment that allows tumor growth and spread. They can also suppress the immune response to tumors and secrete enzymes that break down the extracellular matrix, making it easier for cancer cells to spread. Finally, TAMs physically interact with cancer cells to promote their migration and invasion (18).

In the current study, we detected strong downregulation of ESRP1 and ESRP2 in BC in public database of BC specimens. Moreover, analysis on our own specimens also showed strong downregulation of ESRP1 and ESRP2 in BC, and this downregulation was more pronounced in cases with lung metastasis. *In vitro*, altered ESRP1 or ESRP2 levels caused a switch of alternative splicing of FGFR2 between FGFR2-IIIb and FGFR2-IIIc, resulting in changes in tumor cell growth and metastatic potential. *In vivo*, increased ESRP1 or ESRP2 levels in BC cells not only inhibited the growth of the xenografted tumor formation in nude mice, but also reduced the occurrence of lung metastasis, partially through altering polarization of tumor-associated macrophages.

## Material and methods

### Protocols, patient specimens and experimental design

This study received the official approval from the Ethics Committee of the Shanghai General Hospital. Informed consent was obtained from the patients involved in this study. The BC tissues and normal bladder tissues (NT) were taken from cystectomy. The legitimacy of the observed effects was guaranteed by conducting power calculations (with a significance level of p<0.05) for each experiment to determine the appropriate number of animals to include. To ensure random assignment of experimental units to either the control or treatment group, an allocation concealment technique was employed. By using inbred littermate mice in a specific experiment, the potential for confounding factors was reduced. No animals or experimental units were eliminated during the experiment and all data was included in the analysis.

### Cell culture and transfection

Human BC cell lines T24 and RT4 were both purchased from ATCC (American Type Culture Collection, Manassas, VA, USA). T24 originated from an 81-year-old female Caucasian (20), and RT4 was generated from a 65-year-old male Caucasian (21). Both lines were cultured in Dulbecco’s Modified Eagle Medium (DMEM, Invitrogen, Shanghai, China), supplemented with 10% fetal bovine serum (FBS, Invitrogen) and 1% penicillin/streptomycin (Invitrogen). The plasmids used in this study were obtained from Origene (Beijing, China) and were transfected into cells using Lipofectamine 2000 (Invitrogen). The amount of plasmid used was 1 µg, which had been previously optimized for our experimental conditions. Cells with a confluency of 70% to 80% were selected for transfection, and successful transfection was confirmed by the expression of the GFP reporter in over 90% of the cells.

### *In vivo* transplantation of BC cells and bioluminescence imaging

After subcutaneous injection of 10^6^ cells into nude mice, tumor growth was monitored and quantified over a period of one month using luminescence levels. The luminescence measurements were obtained using the IVIS imaging system (Xenogen Corp., Alameda, CA, USA), which is capable of non-invasive imaging of biological processes in live animals. In addition to monitoring tumor growth, bioluminescence imaging was also utilized to assess the occurrence of lung metastasis. The resulting images were analyzed and quantified using the Living Image software from Xenogen Corp., which allows for accurate measurement of bioluminescence signal intensity. This approach provided a sensitive and non-invasive means of detecting lung metastasis in the experimental animals.

### RNA extraction and quantitative real-time polymerase chain reaction

RNA extraction and RT-qPCR were performed as follows: RNA was extracted using a RNeasy Kit from Qiagen in Shanghai, China. RT-qPCR was done using a SYBR Green PCR Kit from Qiagen and commercially designed primers, also from Qiagen. The RT-qPCR reactions were repeated twice. Gene expression levels were quantified using the 2-△△Ct method, with results presented as relative values after normalization with reference to GAPDH and experimental controls.

### Measurement of cell growth and invasion

Cell growth was assessed with a CCK-8 assay (Roche, Indianapolis, IN, USA). Cell migration was measured by a transwell cell migration assay, a commonly used laboratory technique to study the ability of cells to move through a porous membrane. This assay was typically performed to assess the migratory properties of cells, such as their capacity to invade surrounding tissues, or their response to various stimuli. Briefly, cells were seeded in the top chamber of a transwell insert which was separated from the bottom chamber by a porous membrane. The cells migrated to the bottom chamber containing a chemoattractant that promoted cell migration. After 12 hours, the cells that had migrated to the bottom side of the membrane were fixed, stained with crystal violet. The number of invasive cells was quantified by counting the number of cells that had penetrated through the Matrigel-coated membrane and stained with crystal violet. The relative number of invasive cells was determined by comparing the number of invasive cells in experimental samples with control samples.

### Flow cytometry

For quantification of GFP+ cells in circulation, mouse blood was withdrawn from the tail and subjected to flow cytometry analysis based on direct fluorescent GFP in Becton flow cytometry machine (BD Biosciences, Shanghai, China). The number of the positive cells was automatically obtained by machine counting. For isolation of macrophages in the tumor, dissected tumor was digested with 0.25% Trypsin (Roche) for 30 minutes before all dissociated cells were incubated with PE-conjugated anti-F4/80 antibody (BD Biosciences) for 15 minutes to label all macrophages for sorting.

### Immunostaining

Immunocytochemistry and immunohistochemistry were done with primary antibodies including rabbit anti-ESRP1 and anti-ESRP2 (1:100; Abnova, Taiwan). The secondary antibody used a either cy3- or HRP-conjugated anti-rabbit antibody (1:500; Jackson ImmunoResearch Labs, West Grove, PA, USA). For immunocytochemistry, cultured cells were fixed with 4% paraformaldehyde and then incubated with the ESRP1/ESRP2-specific primary antibody followed by a secondary antibody conjugated with a fluorescent tag, cy3, and observed under a fluorescence microscope. DAPI was used to stain nuclei. DAB staining used a specific kit (Vector Laboratories, Inc. Burlingame, CA, USA).

### Analysis on FGFR2 splicing

The analysis of FGFR2 splice variants was carried out through an RT-PCR protocol that involved the use of AvaI or HincII enzymes to specifically digest exon IIIb- and exon IIIc-containing products. This protocol has been previously described (22). Undigested PCR products were labeled as “Un”. The percentage of exon IIIb inclusion was determined by calculating the ratio of the exon IIIb product to the total of both exon IIIb and exon IIIc products.

### Bioinformatics and statistical analysis

In this study, the public database GSE133624 from the Gene Expression Omnibus (GEO) was utilized (23). To identify differentially expressed genes (DEGs), the GEO2R online tool was employed and the P-value, adjusted P-value, and logFC were calculated. The DEGs underwent pathway enrichment analysis through Metascape (24). Statistical analysis was performed using GraphPad Prism 6 (GraphPad, Chicago, IL, USA) with one-way ANOVA to compare different groups. Post-hoc tests were performed following one-way ANOVA to determine which specific groups differ significantly from one another. Results were expressed as the mean ± standard deviation (SD) and considered significant when p<0.05.

## Results

### Analysis on public database shows downregulation of ESRP1 and ESRP2 in BC

**Figure 1:**
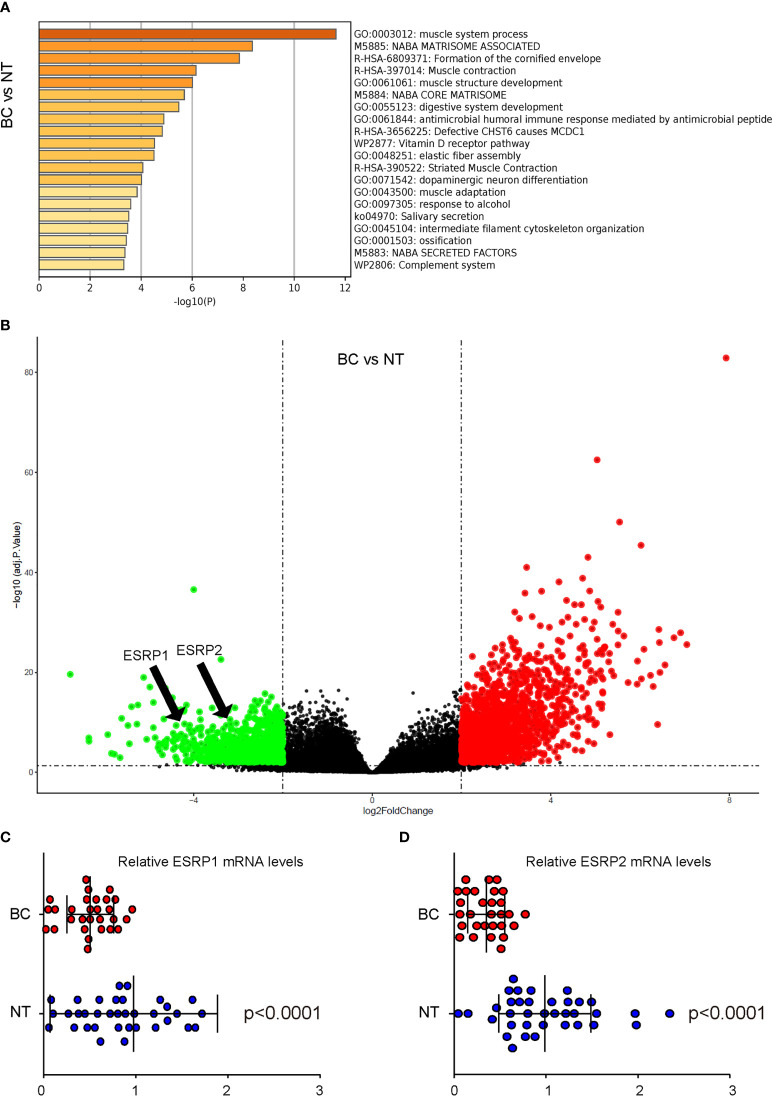
Analysis on public database shows downregulation of ESRP1 and ESRP2 in BC **(A, C)** BC Data from public database GSE133624 were analyzed by an online tool GEO2R. **(A)** Pathway enrichment analysis were assessed by a Metascape online tool **(B)** A volcano map to show that both ESRP1 and ESRP2 were downregulated in BC, compared to NT. **(C, D)** The individual mRNA values for ESRP1 **(C)** and ESRP2 **(D)**. N=33.

ESRPs have been shown to regulate the alternative splicing of FGFR2 to affect EMT-associated cancer cell metastasis in some types of cancers. To assess whether ESRP1/2 may also play a role in the carcinogenesis and lung metastasis in BC, which was not previously studied, we used online tool GEO2R to analyze data from public database on BC. A database GSE133624 was selected, since it was a very new database that has provided a decent analysis on the differentially expressed genes (DEGs) between BC and the normal bladder tissue (NT) from the patients. Pathway enrichment analysis by Metascape online tool was applied to identify altered signaling pathway (**Figure 1A**). Among all DEGs, ESRP1 and ESRP2 were found significantly downregulated, shown in a volcano map (**Figure 1B**). The ESRP1 and ESRP2 levels were significantly lower in BC, compared to NT (p<0.0001, **Figures 1C, D**). These data suggest a possible role of ESRP1 and ESRP2 in the carcinogenesis of BC.

### BC with lung metastasis express lower levels of ESRP1 and ESRP2 compared to BC without lung metastasis

**Figure 2:**
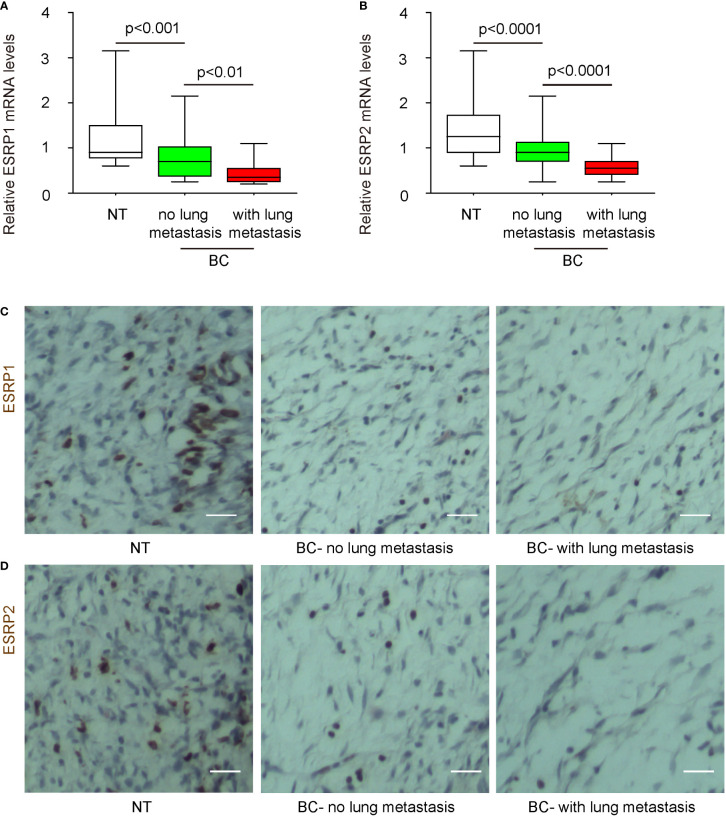
BC with lung metastasis express lower mRNA levels of ESRP1 and ESRP2 compared to BC without lung metastasis. The clinic specimens were catalogized into 3 groups: the specimens from NT (N=50), the specimens from BC without lung metastasis (N=40) and the specimens from BC with lung metastasis (N=12). **(A, B)** RT-qPCR for ESRP1 **(A)** and ESRP2 **(B)** in 3 groups. **(C, D)** Representative immunofluorescent images for ESRP1 **(C)** and ESRP2 **(D)** in tissue from 3 groups. Scale bars are 20µm.

Next, we studied whether ESRP1/2 may also play a role in the lung metastasis in BC. Since there is lack of available data from public database, we analyzed it on the specimens from our own clinic cases. The specimens were catalogized into 3 groups: the specimens from NT (N=50), the specimens from BC without lung metastasis (N=40) and the specimens from BC with lung metastasis (N=12). We found that the levels of ESRP1 and ESRP2 were significantly lower in specimens from BC without lung metastasis, compared to NT. Moreover, the levels of ESRP1 and ESRP2 were significantly lower in the specimens from BC with lung metastasis, compared to the specimens from BC without lung metastasis (**Figures 2A, B**). The expression of ESRP1 and ESRP2 was also shown in the representative immunofluorescent images from 3 groups (**Figures 2C, D**). Together, these data suggest that BC with lung metastasis express lower levels of ESRP1 and ESRP2 compared to BC without lung metastasis.

### Alteration in ESRP1/2 levels in BC cells changes BC cell growth and migration

**Figure 3:**
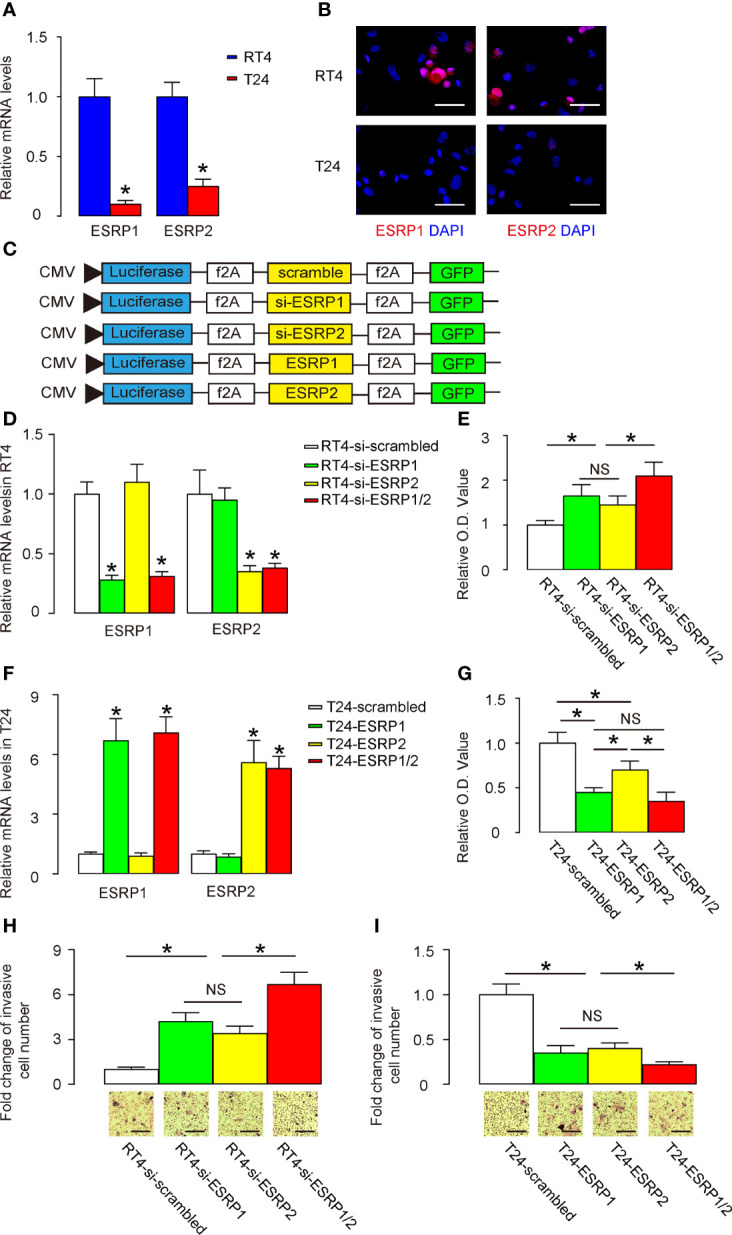
Alteration in ESRP1/2 levels in BC cells changes BC cell growth and migration. **(A, B)** RT-qPCR **(A)** and immunocytochemistry **(B)** for ESRP1 and ESRP2 in RT4 and T24 cells. **(C)** Several plasmids that alter the expression levels of ESRP1 and ESRP2 were prepared. These plasmids were siRNA for ESRP1 (si-ESRP1), siRNA for ESRP2 (si-ESRP2), complete coding sequence for ESRP1 (ESRP1), complete coding sequence for ESRP2 (ESRP2) and a control scrambled sequence (scrambled). These transgenes were under control of a CMV promoter. Two reporters, luciferase and GFP were co-expressed, in order to trace transfected cells *in vivo* by luciferase assay and by fluorescence, respectively. **(D)** RT-qPCR for ESRP1 and ESRP2 in transfected RT4 cells. **(E)** A CCK-8 assay for transfected RT4 cells. **(F)** RT-qPCR for ESRP1 and ESRP2 in transfected T24 cells. **(G)** A CCK-8 assay for transfected T24 cells. **(H, I)** Cell migration was examined in a transwell cell migration assay in transfected RT4 **(H)** and T24 **(I)** cells. *p<0.05. NS, non-significant. N=5. Scale bars are 100µm.

Next, we used BC cells to assess the effects of ESRP1/2 on their growth potential as well as on their migration that is related to lung metastasis. We selected two commonly used BC cell lines, RT4 and T24. While RT4 is a non-invasive benign cell line, T24 is an invasive malignant cell line. Consistent with our findings in patients’ specimens and public database, we found that RT4 expressed significantly higher levels of ESRP1 and ESRP2, compared to T24, by RT-qPCR (**Figure 3A**) and by representative immunofluorescent images of the cultured cells (**Figure 3B**). Thus, we prepared several plasmids that alter the expression levels of ESRP1 and ESRP2. These plasmids were siRNA for ESRP1 (si-ESRP1), siRNA for ESRP2 (si-ESRP2), complete coding sequence for ESRP1, complete coding sequence for ESRP2 and a control scrambled sequence (scrambled). These transgenes were under control of a CMV promoter. Two reporters, luciferase and GFP, were co-expressed in order to trace transfected cells *in vivo* by luciferase assay and by fluorescence, respectively (**Figure 3C**). We found that transfection with si-ESRP1 significantly decreased the levels of ESRP1 without altering ESRP2 levels in RT4 cells, while transfection with si-ESRP2 significantly decreased the levels of ESRP2 without altering ESRP1 levels in RT4 cells. Moreover, no synergic effects were detected on the ESRP1 and ESRP2 levels in RT4 cells co-transfected with si-ESRP1 and si-ESRP2 (**Figure 3D**). These data confirmed the quality and specificity of the prepared plasmids. Furthermore, either transfection with si-ESRP1 or transfection with si-ESRP2 significantly increased the growth potential of RT4 cells in a CCK-8 assay, while combined transfection with both si-ESRP1 and si-ESRP2 exhibited a further increase in RT4 cell growth (**Figure 3E**). In a parallel experiment with T24 cells, we found that transfection with ESRP1 significantly increased the levels of ESRP1 without altering ESRP2 levels, while transfection with ESRP2 significantly increased the levels of ESRP2 without altering ESRP1 levels. Moreover, no synergic effects were detected on the ESRP1 and ESRP2 levels in T24 cells, when the cells were co-transfected with ESRP1 and ESRP2 (**Figure 3F**). These data confirmed the quality and specificity of the prepared plasmids. Furthermore, either transfection with ESRP1 or transfection with ESRP2 significantly decreased the growth potential of T24 cells in a CCK-8 assay, while the effects of ESRP1 appeared to be more pronounced than ESRP2, and a combined transfection with both ESRP1 and ESRP2 did not further decrease the T24 cell growth (**Figure 3G**). The effects of alterations in ESRP1 and ESRP2 levels on cell migration were examined in a transwell cell migration assay. We found that either transfection with si-ESRP1 or transfection with si-ESRP2 significantly increased the migratory RT4 cells, while combined transfection with both si-ESRP1 and si-ESRP2 exhibited a further increase in migratory RT4 cells (**Figure 3H**). On the other hand, either transfection with ESRP1 or transfection with ESRP2 significantly decreased the migratory T24 cells, while combined transfection with both ESRP1 and ESRP2 exhibited a further decrease in migratory T24 cells (**Figure 3I**). Together, these data suggest that alteration in ESRP1/2 levels in BC cells changes BC cell growth and migration, which implies a relationship between ESRP1/2 and lung metastasis.

### Regulation of BC cell invasiveness by ESRP1/2 may be through shifting the epithelial splice form of FGFR2 IIIb to the mesenchymal splice form of FGFR2 IIIc

**Figure 4:**
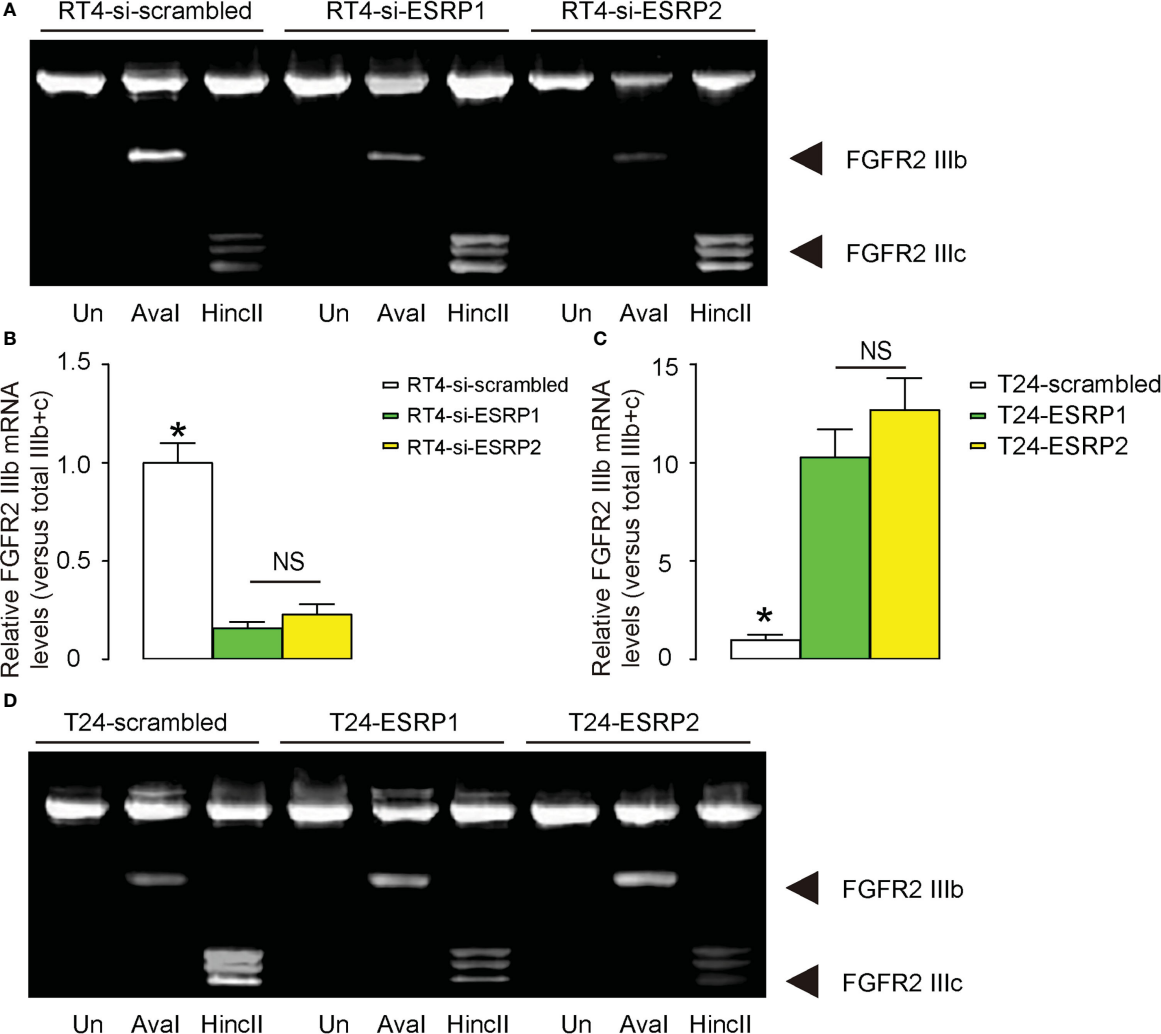
Regulation of BC cell invasiveness by ESRP1/2 may be through shifting the epithelial splice form of FGFR2 IIIb to the mesenchymal splice form of FGFR2 IIIc. **(A, B)** The levels of FGFR2 IIIb and FGFR2 IIIc mRNA in Aval and HincII-digested PCR product for FGFR2 in transfected RT4 cells, shown by representative gel **(A)**, and by quantification of the ratio of FGFR2 IIIb to the combined FGFR2 IIIb and FGFR2 IIIc as the levels of the epithelial versus mesenchymal splicing of FGFR2 **(B)**. **(C, D)** The levels of FGFR2 IIIb and FGFR2 IIIc mRNA in Aval and HincII-digested PCR product for FGFR2 in transfected T24 cells, shown by quantification of the ratio of FGFR2 IIIb to the combined FGFR2 IIIb and FGFR2 IIIc **(C)**, and by representative gel **(D)**. *p<0.05. NS: non-significant. N=3.

A previous study has shown that ESRP1/2 induces the shift from the epithelial splice form of FGFR2 IIIb to the mesenchymal splice form of FGFR2 IIIc, which is important for cancer cell invasion and metastasis (22). To investigate whether this mechanism may also be functional in BC, we analyzed the levels of FGFR2 IIIb and FGFR2 IIIc in Aval and HincII-digested PCR product for FGFR2 in transfected RT4 cells and T24 cells. The ratio of FGFR2 IIIb to the combined FGFR2 IIIb and FGFR2 IIIc was used for quantification of the levels of the epithelial versus mesenchymal splicing of FGFR2. We found that either transfection with si-ESRP1 or transfection with si-ESRP2 significantly decreased the ratio of FGFR2 IIIb versus combined FGFR2 IIIb and FGFR2 IIIc, suggesting a mesenchymal lineage splicing, shown by representative gel (**Figure 4A**), and by quantification (**Figure 4B**). On the other hand, either transfection with ESRP1 or transfection with ESRP2 significantly increased the ratio of FGFR2 IIIb versus combined FGFR2 IIIb and FGFR2 IIIc, suggesting an epithelial lineage splicing, shown by quantification (**Figure 4C**), and by representative gel (**Figure 4D**). Thus, the regulation of BC cell invasiveness by ESRP1/2 may be through shifting the epithelial splice form of FGFR2 IIIb to the mesenchymal splice form of FGFR2 IIIc.

### ESRP1/2 reduces growth and lung metastasis of xeno-transplanted BC cells in nude mice

**Figure 5:**
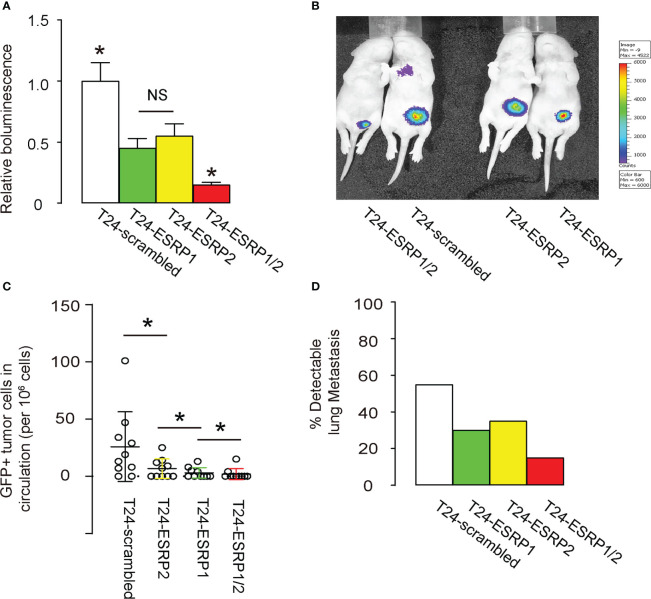
ESRP1/2 reduces growth and lung metastasis of xeno-transplanted BC cells in nude mice. **(A-D)** Equal number of ESRP1, ESRP2, combined ESRP1/2 and scrambled transfected T24 cells were subcutaneously transplanted into the location above the lower abdomen of the nude mice. On month after transplantation, tumor formation in 5 mice was assessed by bioluminescence, shown by quantification **(A)**, and by representative images **(B)**. **(C)** Quantification of GFP+ tumor cells in mouse circulation at 1 month after transplantation. **(D)** The frequency for forming metastatic lung tumors in 20 mice per group. *p<0.05. NS, non-significant.

Finally, equal amounts of ESRP1, ESRP2, a combination of ESRP1/2 and scrambled controls plasmids were transfected into T24 cells. These transfected cells were then subcutaneously transplanted above the lower abdomen of nude mice. After one month, tumor formation was assessed using bioluminescence imaging, which utilized the luciferase expression in the transfected T24 cells that had been transplanted to the nude mice. Significantly smaller tumors were observed in mice grafted with either ESRP1 or ESRP2, compared to scrambled-grafted mice, while the reduction in tumor was even more pronounced in mice grafted with T24 cells transfected with combined ESRP1/2, shown by quantification (**Figure 5A**), and by representative images (**Figure 5B**). Moreover, we also examined GFP+ tumor cells in the circulation and the capability of their circulated tumor cells to develop metastatic lung tumor at 4 weeks after transplantation. The presence of GFP+ cells in circulation was examined by flow cytometry, showing significant reduction in circulating GFP+ cells in mice grafted with ESRP2-transfected T24 cells, compared to those grafted with scrambled-transfected T24 cells. Also, these was a significant reduction in circulating GFP+ cells in mice grafted with ESRP1-transfected T24 cells, compared to those grafted with ESRP2-transfected T24 cells, and a significant reduction in circulating GFP+ cells in mice grafted with ESRP1/2-transfected T24 cells, compared to those grafted with ESRP1-transfected T24 cells (**Figure 5C**). Also, the frequency for the forming metastatic lung tumors was reduced by ESRP1, ESRP2 and ESRP1/2 (**Figure 5D**). Together, these data suggest that ESRP1/2 reduces growth and lung metastasis of xeno-transplanted BC cells in nude mice.

### ESRP1/2 re-expression polarizes TAMs to an anti-tumor phenotype

**Figure 6:**
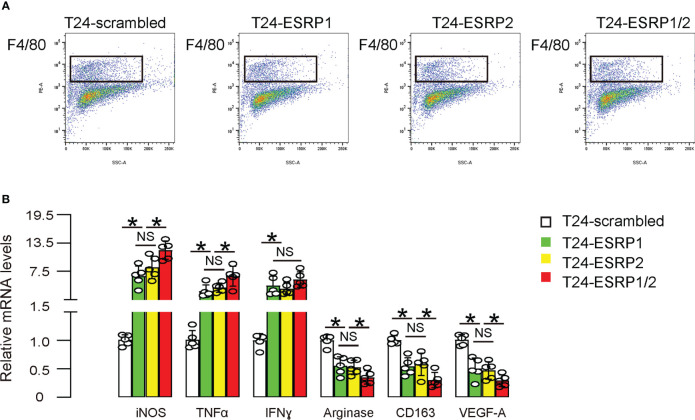
ESRP1/2 re-expression polarizes TAMs to an anti-tumor phenotype. **(A)** F4/80+ macrophages were isolated from the ESRPs-tumors by flow cytometry. **(B)**. RT-qPCR for proinflammatory macrophage-associated genes (iNOS, TNFα and IFNγ) and anti-inflammatory macrophage-associated genes (Arginase, CD163, VEGF-A) in purified macrophages from tumors. *p<0.05. NS, non-significant. N=5.

Since ESRPs-mediated FGFR2 alternative splicing has been shown to alter macrophage polarization in non-tumor models, we thus examined whether the phenotype of TAMs in these ESRPs-treated mice could be altered. F4/80+ macrophages were thus isolated from the ESRPs-tumors (**Figure 6A**). We detected significantly higher proinflammatory macrophage-associated genes (iNOS, TNFα and IFNγ) in purified macrophages from tumors, while significantly lower anti-inflammatory macrophage-associated genes (Arginase, CD163, VEGF-A) in purified macrophages from tumors (**Figure 6B**). These data suggest that ESRP1/2 re-expression polarizes TAMs to an anti-tumor phenotype, which could partially contribute to the anti-metastatic effect of ESRP1/2 in BC *in vivo*.

## Discussion

Previous studies have demonstrated the need of regulatory alternative splicing factors in normal development and a variety of physiological and pathological events (22, 25). Specifically, the alternative splicing of FGFR2 has been shown to be required for a proper regulation of epithelial versus mesenchymal cells. In this process, the expression of FGFR2 pre-mRNA is distinct between epithelial and mesenchymal cells, as it results in the expression of either the FGFR2-IIIb or FGFR2-IIIc isoform, respectively (26, 27). Intriguingly, this unique expression of the FGFR2-IIIb and FGFR2-IIIc isoforms in epithelial versus mesenchymal cells likely represents a specific mechanism that regulates the EMT process, which is the key step for cancer outgrowth and metastasis (14).

The lung metastasis is a severe and relatively rare situation in the muscle-invasive BC, which represent about 30% of all diagnosed cases of BC in the past. However, there are also cases of lung metastasis not stemmed from muscle-invasive BC (28). Since here we showed importance of suppression of ESRP1 and ESRP2 on the BC invasiveness and metastasis, it may be interesting to do a retrospective investigation on these cases to see whether ESRP1 and ESRP2 levels were greatly decreased.

Previous studies have shown the dynamic changes in the alterative splicing of FGFR2-IIIb/IIIc in the cellular transformation during EMT, and its importance in carcinogenesis and tumor metastasis in a variety of cancers (10–13). Nevertheless, this question has not been addressed in BC previously. Here we showed that the depletion of ESRP1 and ESRP2 in a non-invasive BC cell line RT4 rendered the cells from epithelial-like to mesenchymal-like, increased cell growth potential and increased cell invasiveness, likely through a substantial switch of the FGFR2 pre-mRNA from the epithelial IIIb form to the mesenchymal IIIc isoform. On the other hand, the re-expression of ESRP1 and ESRP2 in an invasive BC cell line T24 rendered the cells from mesenchymal-like to epithelial-like, decreased cell growth potential and decreased cell invasiveness, also likely through a substantial switch of the FGFR2 pre-mRNA from the mesenchymal IIIc isoform to the epithelial IIIb isoform. These findings are consistent with the previous reports on the effects of ESRP1 and ESRP2 on EMT (22, 25). Most importantly, the re-expression of ESRP1 and ESRP2 in the invasive T24 BC cells significantly decreased the presence of circulating tumor cells and the occurrence of the lung metastasis.

It is also noteworthy that although we focused on the alternative splicing of FGFR2 pre-mRNA by ESRP1 and ESRP2, the ESRPs do have other targets that may be also involved in the process of EMT related to lung metastasis of BC. However, previous studies have shown that these ESRP targets are enriched for genes that determine the epithelial cell properties, e.g. cytoskeletal reforming, control of cell motility, buildup of cell-cell junctions, and the pathways that regulate EMT (22, 25). These reports suggest that ESRP-regulated alternative splicing may regulate a number of factors to coordinate the control of EMT during lung metastasis of BC. Our study indicates ESRP1/2 as promising novel targets to suppress BC invasiveness and malignant metastasis in the therapy.

The effects of ESRP1/2 on FGFR2 splicing may not only occur in BC cells, but also occur in TAMs, since TAMs are known to express very high levels of FGFR1 and FGFR2 (17). The clinic samples did not distinguish tumor cells from intratumor non-tumor cells, such as macrophages. Thus, it is highly possible that this regulatory axis (ESRP1/2-FGFR2) may also regulate TAM functionality and phenotypic adaption, which was supported by the data from the current study. It may be interesting to further assess it in tumor cells and TAMs separately and interactively to further our knowledge of its role in BC metastasis.

## Data availability statement

The original contributions presented in the study are included in the article/supplementary material. Further inquiries can be directed to the corresponding authors.

## Ethics statement

The studies involving human participants were reviewed and approved by Shanghai General Hospital. The patients/participants provided their written informed consent to participate in this study. The animal study was reviewed and approved by Shanghai General Hospital.

## Author contributions

HL is responsible for study conception and design. ML, WM, WW, YZ and HL performed bioinformatics analysis and are responsible for data acquisition and analysis. HL wrote the manuscript and all authors have read the manuscript and agreed with the publication. HL is responsible for funding and are the guarantee of the study. All authors contributed to the article and approved the submitted version.
